# Identification of a variant in *NLRP3* gene in a patient with Muckle-Wells syndrome: a case report and review of literature

**DOI:** 10.1186/s12969-023-00795-x

**Published:** 2023-02-10

**Authors:** Jia Liu, Ranran Zhang, Zhi Yi, Yi Lin, Hong Chang, Qiuye Zhang

**Affiliations:** 1 Deparment of Pediatric Nephrology, Rheumatology and Immunity, The Affiliated Hospital of Qingdao University, Qingdao, China; 2 Deparment of Pediatric Neurology, The Affiliated Hospital of Qingdao University, Qingdao, China

**Keywords:** Cryopyrin-associated periodic syndrome (CAPS), Muckle-Wells syndrome (MWS), *NLRP3* gene, Variation

## Abstract

**Background:**

Cryopyrin-associated periodic syndrome (CAPS), a rare genetic autoimmune disease, is composed of familial cold autoinflammatory syndrome (FCAs), Muckle-Wells syndrome (MWS), and neonatal onset multisystem inflammatory disease (NOMID). MWS is caused by dominantly inherited or de novo gain-of-function mutations in the NOD-like receptor 3 (*NLRP3*) gene. At present, there is no report about the variation of R262W in China.

**Case presentation:**

We reported a 3-year-old Chinese boy who had recurrent fever without obvious inducement, bilateral conjunctival congestion, and urticarial-like rash. Laboratory examination showed elevation in leukocyte count, neutrophil count, erythrocyte sedimentation rate (ESR), and C-reactive protein (CRP) and serum amyloid protein (SAA) levels. Whole exome sequencing identified a missense variation c.784-786delinsTGG (p.R262W) in the coding region of the *NLRP3* gene.

**Conclusion:**

A classical variant of the *NLRP3* gene in a patient with MWS was first reported in China.

## Background

Cryopyrin-associated periodic syndrome (CAPS) is a rare genetic autoimmune disease with clinical heterogeneity. CAPS includes mild, moderate, and severe phenotypes. The mild phenotype is known as familial cold autoinflammatory syndrome (FCAs), the moderate phenotype is known as Muckle-Wells syndrome (MWS), and the severe phenotype is known as neonatal onset multisystem inflammatory disease (NOMID), also known as chronic infantile neurocutaneous joint syndrome [1,2].

CAPS is caused by variations in the *NLRP3* gene, which encodes cryopyrin, a key protein component of inflammasomes. These variations can lead to excessive production of interleukin-1β (IL-1β) which can lead to the occurrence of inflammatory reaction.

Here we reported a case of MWS, who had recurrent fever, urticarial-like rash, and bilateral conjunctival congestion. Whole exome sequencing identified a missense variation c.784-786delinsTGG (p.R262W) in the coding region of the *NLRP3* gene. This gene variation site was the first to be reported in China.

## Case presentation

A 3-year-old boy was first referred to our department in 2020 with complaints of recurrent fever with no apparent cause for more than 10 months. Each episode of fever generally lasted for about 1–5 days, and the interval between each episode was approximately 15 days. During all episodes of fever, the temperature was about 37.5ºC with occasional blips up to 39ºC, accompanied by bilateral conjunctival congestion and urticarial-like rash. There were no fatigue, headache, nasal congestion, runny nose, sore throat, cough and asthma, abdominal pain, vomiting and diarrhea, and joint swelling and pain. Routine blood examination showed elevation in leukocyte count, neutrophil count, erythrocyte sedimentation rate (ESR), and C-reactive protein (CRP) and serum amyloid protein (SAA) levels.

**Fig.1:**
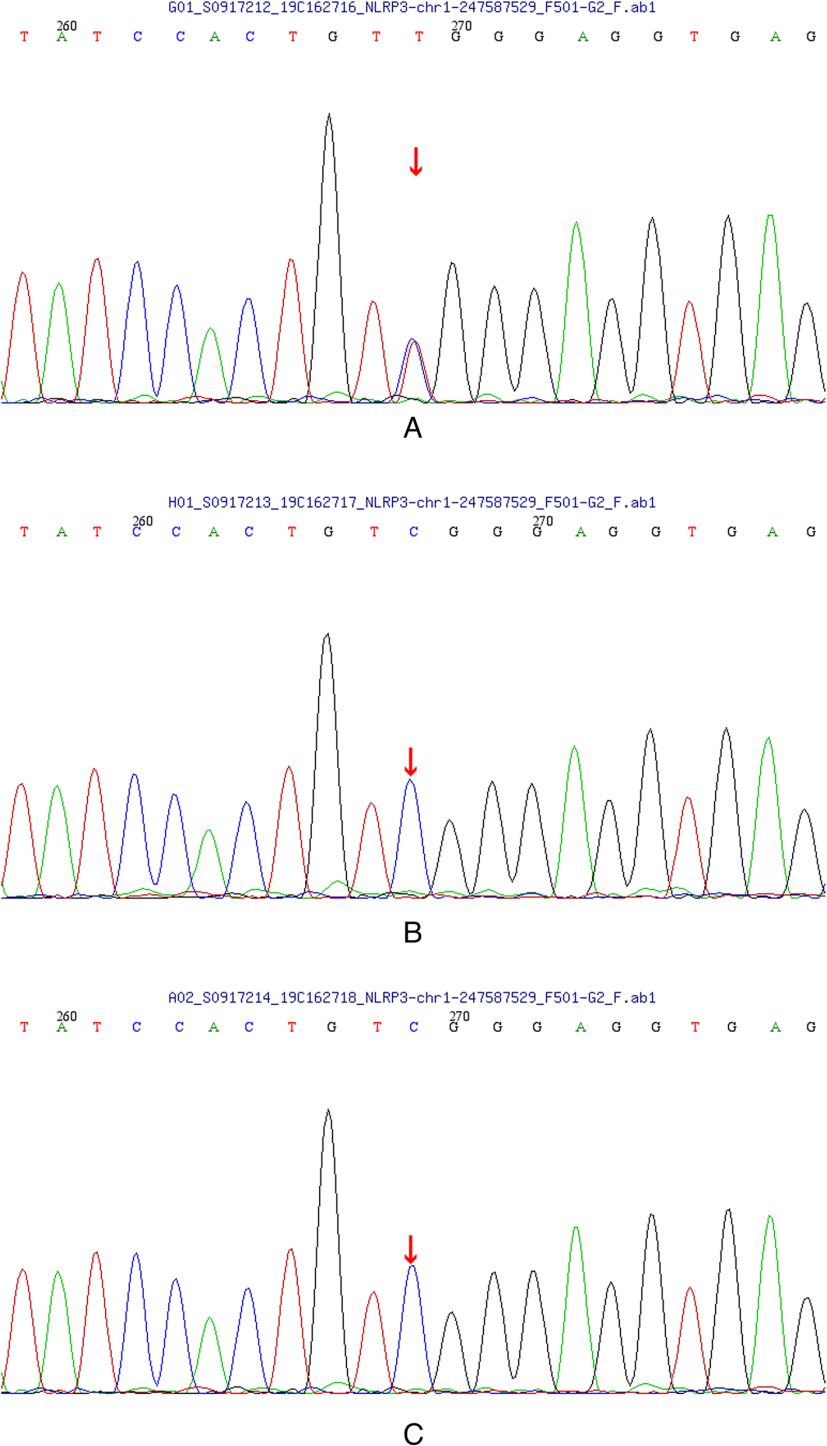
Sanger sequencing of *NLRP3* gene in patient and his parents. **a** The position of the variant in *NLRP3* gene in the patient. **b** The father of the patient has no variation at this locus. **c** The mother of the patient has no variation at this locus

Whole exome sequencing identified a heterozygous variation of *NLRP3* gene (c.784-786delinsTGG) in the coding region that resulted in amino acid substitution of arginine for tryptophan at codon 262 (p.R262W), which is a missense variation. The frequency of variation in this database is normal. Through family verification analysis, the parents of the subject had no variation at nucleotide locus 784 in the coding region, suggesting a spontaneous mutation. According to the ACMG guidelines, this mutation was preliminarily determined to be pathogenic. Furthermore, we verified the gene function through two software, and the results are as follows: 1. This variation is predicted to be probably damaging with a score of 1.000 by PolyPhen-2. 2. This variation is predicted to be disease causing with a score of 0.999 by MutationTaster. The mutation was validated using Sanger sequencing (Fig. 1).

According to clinical manifestation, laboratory examination results, and sequencing results, the boy was diagnosed with Muckle-Wells syndrome. He was subsequently treated with oral thalidomide at 12.5 mg, twice a day. During the medication period, there were no symptoms such as fever, rash, and conjunctivitis.

## Discussion

Autoinflammatory disease (AUID) is caused by gene variations that change the protein encoded by them, leading to innate immune dysfunction and subsequent systemic excessive inflammatory response [3]. Most patients with AUID show sudden periodic fever, rash, serositis, lymphadenopathy, and arthritis. Inflammatory indexes generally increase during the attack period. However, during the asymptomatic interval, the growth and development of the patients are normal, as well as the inflammatory indexes. Among them, AUID caused by single gene variations are called monogenic autoinflflammatory diseases. Due to their genetic characteristics, the onset of this disease is early, and it can occur from newborn to adolescence [4]. With the development of gene sequencing technology, more than 50 monogenic autoinflflammatory diseases have been identified [5].

CAPS is an extremely rare continuum of autoinflammatory disease with severe and persistent inflammation status. This syndrome encompasses a spectrum of three subsets according to different clinical manifestation: FCAS, MWS and CINCA/ NOMID. CAPS was originally considered as three independent diseases. In fact, it is now considered as a disease process with different severity under the same genetic background. The clinical characteristics of CAPS was shown in Table 1. In 1962, Muckle and Wells first described MWS with manifestations of urticaria rash, neurosensory hearing loss, and amyloidosis syndrome [6]. The typical clinical characteristics of MWS include: onset from infancy or early childhood, repeated intermittent fever without specific inducing factors (not closely related to cold exposure), accompanied by urticarial-like rash, joint pain, headache, conjunctivitis, etc., and the elevated systemic inflammatory indexes (leukocyte count, neutrophil count, CRP, SAA, ESR, etc.). The symptoms can last from 1 day to 2 weeks, and the interval between onset varies from a few weeks to several months. Sensorineural deafness can occur during adolescence, and 1/3 of patients will have AA-type amyloidosis, which can cause serious complications such as renal failure [7]. The main manifestations in this case were recurrent fever, urticarial-like rash, and conjunctivitis without obvious inducement, accompanied by elevated neutrophils, CRP, SAA, and ESR. After the administration of thalidomide, the symptoms were well controlled, and the laboratory indexes returned to normal. The MWS-DAS score of this case was less than 10 [8,9]. Considering that the disease activity was mild, long-term follow-up is needed in the future to pay attention to the occurrence of hearing damage, AA amyloidosis, and kidney damage.

**Table 1:** Clinical characteristics of CAPS (adapted from [10,11])

	FCAS	MWS	CINCA/NOMID
Severity	mild	moderate	severe
Age at onset	< 6 months-adulthood	Early childhood-adulthood	Perinatal
Family history	Often positive	Often positive	Often negative
Triggers	Generalized cold/pneumovax	Stress/excercise/infection/pneumovax	Stress/excercise/infection/pneumovax
Cold trigger	Yes	Possible	Rare
Fever	Yes	Yes	Yes
Fatigue	Rare	Yes	Yes
Cutaneous manifestations	Urticaria-like rash	Urticaria-like rash	Urticaria-like rash
Ocular manifestations	Conjunctivitis/keratitis	Conjunctivitis/ketatitis/uveitis	Conjunctivitis/ketatitis/uveitispapilledema
Muskulosceletal manifestations	Myalgia/arthralgia	Myalgia/arthralgia/arthritis	Myalgia/arthralgia/arthritis/distal femur overgrowth
Central nervous system manifestations	Headache	Headache/intermittent aseptic meningitis	Headache/sterile meingtitis/elevated intracranial pressure/brain atrophy
Hearing loss	No	Yes	Yes
Amyloidosis	Rare	Yes	Yes

CAPS is caused by dominantly inherited or de novo gain-of-function mutations in the NOD-like receptor 3 (*NLRP3*) (also known as *CIAS1* or *NALP3*) gene located on chromosome 1q44 [12]. The *NLRP3* gene encodes the NLRP3 protein (cryopyrin), which can oligomerize and recruit other intracellular proteins such as ASC (apoptosis related spot like protein containing caspase recruitment domain) and several chaperones to form NLRP3 inflammasomes. The formation of this complex activates the proinflammatory protease called caspase-1, which in turn mediates release of proinflammatory cytokines such as IL-1β [13–16]. IL-1β can cause fever, vasodilation, and systemic inflammation. Therefore, *NLRP3* gene variation can lead to continuous activation of inflammasomes and excessive production of IL-1, leading to systemic inflammation [17,18]. The incidence rate of CAPS in the United States is about 1/1,000,000 [19], and the estimated prevalence in France is 1/360,000 [20]. However, the incidence rate in China is still unknown. In 2012, the expert group developed a set of practice guidelines for hereditary recurrent fever (HRF), which pointed out some specific gene variation sites of CAPS (p.R260W, p.D303N, p.L305P, p.E311K, p.T348M, p.L353P, p.A439V, p.V198M, and p.Q703K) [21]. Infevers database (https://infevers.umai-montpellier.fr/web/) listed more than 240 sequence variants of *NLRP3* gene as of November 2020, and more than 100 are known to be pathogenic/possible pathogenic, and most of which are located in exon 3 [22]. In recent years, with deepened understanding of the diseases and widespread application of second-generation sequencing technology, many cases of CAPS have been reported in China [23–26]. The R260W (also known as R262W) variation of *NLRP3* gene was first reported in 2002 and its involvement in MWS and FCU was confirmed, but some clinical symptoms, such as deafness, AA-type amyloidosis, and cold sensitivity, do not always exist, indicating that *NLRP3* gene variation is not an independent pathogenic factor, and there may be epigenetic and other factors involved in the pathogenesis. Unknown gene modification may affect its phenotype [27]. Our sequencing results showed that there was a heterozygous variation in the *NLRP3* gene, which led to the R > W missense variation at amino acid 262. Considering his pathogenesis, clinical and laboratory manifestations, he was diagnosed with MWS. Previous studies have found that MWS is common in the Nordic population. Our findings have expanded the ethnic scope and clinical manifestation spectrum of the disease. The variation site of R260W was reported for the first time in China.

It has been reported that the clinical phenotypes of MWS patients with the variation of R260W mainly include: symptoms after 6 months (median > 2 years), positive family history, cold trigger attack, and most of them have experienced chronic disease course [2,28]. This case showed symptoms at the age of 3 years and experienced a chronic course of disease, which seems to be inconsistent with previous literature reports. In combination with other MWS cases reported in China so far (Table 2), we found that the clinical manifestations of MWS cases in China were basically consistent with those reported abroad, but no renal amyloidosis has occurred so far, which may be related to the short follow-up time and small number of cases, and also not exclude the relationship with race, epigenetic and other factors.

**Table 2:** Summary of the patients with MWS reported in China

Patient	Gender	Age at onset (years)	Family history	Cold trigger	Clinical characteristics										
					Fever	Skin rash	Oral ulcer	Conjunctivitis	Myalgia	Arthralgia/arthritis	Abdominal pain	Headache	Hearing loss	Chronic meningitis	Renal amyloidosis
1^[[[23]]]^	Male	7	×	×	√	√	×	×	×	√	×	×	×	×	×
2^[[[23]]]^	Male	46	×	×	√	√	√	×	√	√	√	×	×	×	×
3^[[[23]]]^	Male	2	×	×	√	√	√	√	√	×	×	√	×	×	×
4^[[[23]]]^	Male	2	×	×	√	√	√	√	×	√	×	√	√	√	×
5^[[[24]]]^	Male	7	×	×	√	√	×	×	×	√	×	×	×	×	×
6^a^	Male	3	×	×	√	√	×	√	×	×	×	×	×	×	×

Patient	Laboratory indicators			*NLRP3* variation		Treatment
	Increased CRP	Increased ESR	Increased SAA	Nucleotide exchange	Amino acid exchange	
1^[[[23]]]^	√	√	-	c.86A > T	p.D26V	prednisone + MTX
2^[[[23]]]^	√	√	-	c.2107C > A	p.Q703K	prednisone + TwHF
3^[[[23]]]^	√	√	-	c.208G > A	p.V70M	prednisone
4^[[[23]]]^	√	√	-	c.1043C > T	p.T348M	prednisone + CsA
5^[[[24]]]^	√	√	-	c.92A > T	p.D31V	prednisone + MTX → colchicine → etanercept → ×
6^a^	√	√	√	c.784-786delinsTGG	p.R262W	thalidomide

^a^Case in this report

The objective of the treatment of CAPS is to inhibit systemic inflammation, prevent organ injury, and improve the quality of life of patients. Due to the central role of IL-1 in the pathogenesis of CAPS, biologically targeted therapy against IL-1 is recommended for CAPS [29]. At present, three IL-1 blockers consisting of anakina, canakinumab, and rilonacept are approved for CAPS. Anakinra is a short acting recombinant IL-1 receptor antagonist, which has been approved by the European Drug Administration (EMA) and the U.S. Food and Drug Administration (FDA). Canakinumab is an EMA and FDA approved fully humanized monoclonal antibody against IL-1, which can selectively bind soluble IL-1. Rilonacept is a soluble recombinant IL-1 receptor antagonist, which has only been approved by FDA. It is a pity that it is difficult to purchase IL-1 blockers from abroad due to COVID-19. And the domestic IL-1 blockers are still in the clinical trial stage. In addition, some non-steroidal anti-inflammatory drugs and immune modulators have been confirmed that they play a role in improving symptoms. Thalidomide was used to treat pregnancy vomiting in the 1950s, and was later stopped because of the deformity of newborn limbs [30]. Later, it was accidentally discovered that thalidomide can be used to treat leprosy erythematous nodules. Its anti-inflammatory, immunosuppressive and anti-tumor effects have gradually attracted widespread attention, and it is now used to treat multiple myeloma, leukemia, systemic lupus erythematosus, inflammatory bowel disease, Behcet's disease, adult still's disease and other diseases [31,32]. Furthermore, it has been reported that the clinical course of CAPS patient was improved during and after treatment with thalidomide [33].

The immunoregulatory effect of thalidomide is mainly manifested in the expression of some cytokines and adhesion molecules, as well as the regulation of immune cell activity: 1. Regulatory effect on cytokines. (1) Regulation of TNF-α: thalidomide can specifically reduce the level of TNF-a and regulate the secretion of other cytokines induced by TNF-α (such as IL-6, 8, 12, etc.). The regulation of thalidomide on TNF-α may be through the regulation of cytochrome C pathway, leading to apoptosis of monocytes, accelerating the degradation of TNF-α mRNA, thus leading to the decrease of TNF-α production, and thus affecting the inflammatory response [34,35]. (2) Regulation of IL: thalidomide can effectively inhibit the production of IL-1, 6 and 12, increase the production of IL-2, 4 and 10, and thus reduce inflammation. Thalidomide has a regulatory effect on lymphocytes, which is shown in that it can stimulate the proliferation of CTL cells and reduce the ratio of CD4 + /CD8 + . The effect of thalidomide on IL may be through changing the expression of ICAM-1 and LFA-1 on the surface of peripheral blood cells, affecting the interaction between cells, reducing the aggregation of lymphocytes to inflammatory sites, and thus regulating the production of IL [36]. It has also been found that thalidomide can play a role by inhibiting the expression of IL-6 receptor mRNA in a time and dose dependent manner [35]. 2. Inhibition of NF-κB. NF-κB is a key substance regulating inflammatory factor gene, and exists in the form of complex with inhibitor I-κB in the cytoplasm. It was found that thalidomide may selectively inhibit the activation of NF-κB mediated by inflammatory factors by acting on I-κB kinase, inactivate the expression of NF-κB, and then inhibit the expression of related inflammatory factors [37]. 3. Anti angiogenic effect: Thalidomide can significantly inhibit VEGF secretion and angiogenesis, increase cell apoptosis, and its anti angiogenesis effect may be related to the angiointegrin pathway [38]. Therefore, we finally chose thalidomide for empirical treatment in view of the light clinical performance of this patient, combined with the drug source, cost, treatment tolerance and potential side effects of IL-1 blocker. Interestingly, we found that the clinical manifestations and laboratory indicators of this case have been well controlled for 2 years after the empirical administration of thalidomide. At the same time, no side effects related to thalidomide have been found during the follow-up period. We speculated that this might be related to the mild condition of the case, but we also speculated whether it was also relevant to the gene variation of c.784-786delinsTGG (p.R262W), which needs to be further studied in the future. What is more important, close follow-up is still needed to regularly monitor the disease activity and evaluate organ damage in the future.

As we all know, fever is the most common clinical manifestation in children, and the causes are also diverse. Therefore, the diagnosis and differential diagnosis of fever in children is very challenging. Thus, clinically, children with recurrent fever, rash, musculoskeletal symptoms, and elevated inflammatory indicators, especially those with a positive family history, should be alert to the possibility of CAPS. If CAPS is suspected, the NLRP3 gene should be detected using molecular genetics for the presence of variations. If diagnosis is confirmed, the patients should be treated individually and closely followed up to prevent further organ damage and maximize the quality of life.

## Conclusion

In this case, MWS was suspected and confirmed by the presence of a classical variation of the NLRP3 gene, which has also been reported in other MWS patients. This variation was reported in China for the first time. Our findings have expanded the ethnic scope and clinical manifestation spectrum of the disease, and equally important, provide a new idea for the diagnosis of recurrent fever. However, more in-depth follow-up and research are needed to optimize the treatment of MWS.

## Data Availability

Not applicable.

## Abbreviations

CAPS: Cryopyrin-associated periodic syndrome
FCAS: Familial cold autoinflammatory syndrome
MWS: Muckle-Wells syndrome
NOMID: Neonatal onset multisystem inflammatory disease
NLRP3: NOD-like receptor 3
ESR: Erythrocyte sedimentation rate
CRP: Erythrocyte sedimentation rate
SAA: Erythrocyte sedimentation rate
IL-1β: Interleukin-1β
AUID: Autoinflammatory disease
HRF: Hereditary recurrent fever
EMA: European drug administration
FDA: Food and drug administration
MTX: Methotrexate
